# Community based peer counsellors for support of exclusive breastfeeding: experiences from rural Uganda

**DOI:** 10.1186/1746-4358-1-19

**Published:** 2006-10-20

**Authors:** Jolly Nankunda, James K Tumwine, Åshild Soltvedt, Nulu Semiyaga, Grace Ndeezi, Thorkild Tylleskär

**Affiliations:** 1 Department of Paediatrics and Child Health, Makerere Medical School, Kampala, Uganda; 2 Centre for International Health, Armauer Hansen Building, N-5021 Bergen, University of Bergen, Norway

## Abstract

**Background:**

Universal exclusive breastfeeding for the first six months could reduce infant mortality by 13%. Although 99% women initiate breastfeeding in Uganda, exclusive breastfeeding rates remain low. Although peer counsellors for support of breastfeeding mothers have been found useful in other countries, they have not been used in Uganda. The aim of this pilot study was to assess the feasibility of training community based peer counsellors to support exclusive breastfeeding in a rural district in Uganda.

**Methods:**

With assistance of the investigators, the local communities selected fifteen women aged 25 to 30 years. These women were trained for five days on breastfeeding counselling using the La Leche League curriculum. After training they returned to their communities and started supporting breastfeeding peers. They were followed up and supported in their work for three months. The programme was evaluated through focus group discussions with the peer counsellors, fathers and mothers.

**Results:**

The trainees appreciated the knowledge gained and discussed cultural beliefs which affect breastfeeding. They offered breastfeeding support to 15 mothers each within the first two months. They found time to visit and help their breastfeeding peers despite busy schedules. They identified common breastfeeding problems as "insufficient breast milk", sore nipples, breast engorgement, mastitis and poor positioning at the breast. They further observed that most of these problems were eased by correct positioning of the baby at the breast. The peer counsellors were easily accepted by their communities. The mothers were happy to have someone within their community helping them with their breastfeeding problems. Although the peer counsellors were initially selected as volunteers, soon they demanded remuneration.

**Conclusion:**

The training and follow up of peer counsellors to support exclusive breastfeeding in this rural district is feasible. The peer counsellors were accepted by their communities.

## Background

It has been estimated that exclusive breastfeeding for the first six months of life could reduce infant mortality rate by a remarkable 13% [1]. Exclusive breastfeeding has been defined as feeding an infant with breast milk only without giving any other foods, not even water. The definition allows for prescribed medicines, immunisations, vitamins and minerals supplements [2]. The risks of not breastfeeding have been highlighted. These include high infant mortality as a result of reduced protection against certain preventable deaths from infectious [3,4] and possibly also chronic diseases, gastrointestinal infection [5] and delayed recovery from illness. In Dhaka, Bangladesh, it was reported that partial or no breastfeeding was associated a 2.23-fold higher risk of infant deaths from all causes [6].

There are many cultural and practical obstacles to the practice of exclusive breastfeeding. Some traditional beliefs, practices and rites encourage use of pre-lacteal feeds, as well as giving extra water, herbs and "teas" to breastfeeding babies [7-9]. In rural Yoruba communities, exclusive breastfeeding is considered dangerous to the infant who is thought to require water to quench thirst and promote normal development [10]. Many women start mixed feeding because they have to resume work or even return to school [11].

In Uganda, breastfeeding remains a culturally accepted practice with up to 99% of women initiating breastfeeding [12]. However, exclusive breastfeeding rates remain low in the country [12,13]. According to the Uganda Demographic and Health survey 2000–2001, 62% of children under six years of age were exclusively breastfed as compared to 74% of those aged under four months [14]. These figures were based on the 24-hour recall before the survey. The challenge is how to scale up exclusive breastfeeding to universal levels.

An additional problem in Africa over the last two decades has been the potential transmission of HIV through breast milk. A recent study from Zimbabwe indicates that postnatal transmission of HIV can be halved from 14% to 7% by exclusive breastfeeding in the first three months [15]. There is circumstantial evidence that the fear of spreading HIV to their infants through breast milk has scared mothers [16,17], some of whom may not know their HIV sero-status, with resultant negative influences on their breastfeeding practices. On the other hand, recent studies done in Africa have reported that women who are HIV-positive continue to breastfeed to avoid stigmatization by their families and communities [17,18].

Several initiatives to improve exclusive breastfeeding have been tried with varying success. These include: the implementation of Baby Friendly Hospital Initiative (BFHI) recommendations (10 steps to successful breastfeeding – see Table 1) in maternity hospitals, education of mothers on how to breastfeed successfully, paternal support and use of peer counsellors to support breastfeeding mothers [5,19-22]. The purpose of BFHI is to actively protect, promote, encourage and support breastfeeding through education of health care workers in maternity and neonatal services. It also accredits hospitals and maternity units that demonstrate that they meet the WHO/UNICEF criteria as a Baby Friendly Hospital. However, using health workers to give early support for exclusive breastfeeding in Italian women was reported as ineffective [23]. This calls for further research to identify reasons for this finding since it contrasts the findings reported in the Cochrane review of breastfeeding support using 13 trials where provision of extra support to mothers by professionals with special skills in breastfeeding led to increase in the number of mothers exclusively breastfeeding up to two months [24]. Program data from Ghana, Madagascar and Bolivia used several methods which included skills training, harmonised messages and peer group support and interaction to promote breastfeeding in the community [25]. The rates of timely initiation of breastfeeding and exclusive breastfeeding (by 24-hour recall) increased over the three year period of implementation of the program [25].

**Table 1:** Ten Steps to Successful Breastfeeding

Every facility providing maternity services and care for newborn infants should:

1. Have a written breastfeeding policy that is routinely communicated to all health care staff.
2. Train all health care staff in skills necessary to implement this policy.
3. Inform all pregnant women about the benefits and management of breastfeeding.
4. Help mothers initiate breastfeeding within a half-hour of birth.
5. Show mothers how to breastfeed, and how to maintain lactation even if they should be separated from their infants.
6. Give newborn infants no food or drink other than breast milk, unless medically indicated.
7. Practice rooming-in – allow mothers and infants to remain together – 24 hours a day.
8. Encourage breastfeeding on demand.
9. Give no artificial teats or pacifiers (also called dummies or soothers) to breastfeeding infants.
10. Foster the establishment of breastfeeding support groups and refer mothers to them on discharge from the hospital or clinic.

In Uganda, where the majority of women deliver outside health facilities [14], the BFHI strategy alone, being hospital based, would miss out most mothers. One way of improving exclusive breastfeeding levels is through support of breastfeeding mothers by peer counsellors. Peer counselling is an effective way of promoting exclusive breastfeeding [20] and it has also been reported to decrease the speed of weaning [26]. Haider and others reported an improved rate of exclusive breastfeeding at five months of age in a group of mothers supported by peer counsellors (70%) compared to the control group (6%) [20]. In Ghana, lactation counselling was reported to lead to increased exclusive breastfeeding rates using the "previous month" exclusive breastfeeding [27]. Most of the reports on peer counsellors have come from high income countries [19,21,22,28,29], Eastern Europe [5] and Asia [20]. Hitherto there is no experience with the use of peer counsellors for promotion of breastfeeding in Uganda.

This study assessed the feasibility of training community women as peer counsellors for promotion of exclusive breastfeeding, observed their performance as well as their acceptability by their community in Iganga, a rural district in Eastern Uganda.

## Methods

### Study area

This descriptive study was carried out in 2004, in Ibulanku Sub County, Iganga district in eastern Uganda. Ibulanku sub-county is located on the Nairobi highway, 200 km east of Kampala. The sub-county is divided into ten parishes with a population of 38,000 of whom about 3700 are women in the reproductive age group [14]. The sub-county has one health centre with an operating theatre and maternity ward, staffed by a doctor, a clinical officer, nurses and midwives. There are six other smaller health units within the sub-county.

### Selection of peer counsellors

Together with members of the district health team, we sensitised 15 leaders (one from each community) about the study. The community leaders and the research team discussed demographic characteristics of the population and reviewed their records in order to estimate the population of pregnant women. This information was used to estimate the number of peer counsellors to train. Each community then selected one woman to train as a peer counsellor using agreed criteria. To be selected, a woman, had to be aged 24–35 years, a regular resident of the area, and must have breastfed a child who was less than five years old. The women had to be literate in *Lusoga*, the local language, and acceptable to the community. Women were considered acceptable to the community when the community was willing to associate with them by allowing them to visit their homes freely and talk to the mothers. These women also had to be of good repute within their community and not regarded as outcasts. Women with visual and hearing impairment and those who could not attend all sessions of the training were not recruited.

### Training

The training was run by two lactation consultants using the La Leche League 18-hour training curriculum from 29 March to 3 April 2004. One had experience in training peer counsellors in Cape Town, South Africa and the other was a local consultant with experience in training health workers in infant feeding counselling. The training was done in two phases: Training of Trainers (ToT) where eighteen trainers who included a group of policy makers, skilled breastfeeding trainers and others with nursing or medical background were trained for three days and the training of peer counsellors for five days. We utilized lectures and simulated counselling sessions in order to prepare for training of the peer counsellors. We used the Breastfeeding Answer Book by Mohrbacher and Stock as reference material [30].

The training of the peer counsellors was carried out by recently trained trainers under the supervision of lactation consultants. A list of what the peer counsellors wanted to learn about breastfeeding and their expectations was made at the beginning of the training. This was reviewed throughout the training to ensure that their expectations were met. Training methods used included interactive lectures, group discussions, role-plays, and practical sessions for hands-on experience in the hospital antenatal clinic and maternity ward. In addition the participants visited one of the communities to practice peer counselling at community level. The trainers observed the peer counsellors during sessions and asked them questions to check their understanding. They also held meetings every evening to discuss and evaluate the peer counsellors' progress as well as plan the next day's sessions. The peer counsellors practiced filling in a simple record form which they would use on returning home in their respective communities.

A follow-up plan was made by the peer counsellors and the trainers at the end of the training. Each peer counsellor would be visited by a supervisor every two weeks and a monthly meeting held for all.

Seven health workers from the health units in Ibulanku Sub County were taken through a three day training similar to what the peer counsellors had received in preparation for referrals by the peer counsellors.

### Evaluation of peer counselling

After training, the peer counsellors reported to their community leaders and started identifying and recruiting pregnant mothers in the last trimester for counselling on exclusive breastfeeding. Once every two weeks, a supervisor observed each peer counsellor during a counselling session and supported her. Any observed gaps in knowledge and skills were discussed after the counselling session.

The supervisors recorded their observations of the peer counsellors' skills practice. The peer counsellors and supervisors held monthly meetings where reports and challenges by peer counsellors were discussed and possible solutions agreed. Three monthly meetings were held during this study. We used the minutes of these meetings and supervisors' reports to develop impressions about the progress of the peer counselling process.

We held two focus group discussions with peer counsellors: at the start of training and at the end. We also held two focus group discussions with the mothers who were being supported to breastfeed after two months of the peer counselling and two with the men in the community after two months of counselling. Each focus group discussion was moderated by two people with experience in moderating focus group discussions. One led the discussion while another took notes and handled the tape recorder. The outcome of these focus group discussions were used to evaluate the progress of the peer counselling program.

### Data collection and analysis

Data were collected using qualitative methods: participatory observations during selection of peer counsellors and training, key informant interviews of the community leaders and reports from the district supervisors, peer counsellors as well as minutes of monthly meetings with peer counsellors. Focus group discussions were recorded and manually transcribed by three persons with experience working with focus group discussions and one of the authors. The transcripts were used to develop general impressions which were entered onto the analysis form. Significant responses were identified and the emerging themes compiled from which conclusions were made.

### Ethical considerations

Permission to conduct the study was obtained from the Makerere Faculty of Medicine Ethics and Research committee, the Uganda National Council of Science and Technology and the Iganga District administration. The community leaders of Ibulanku sub-county also consented on behalf of their communities to participate in the study.

## Results

### The training

We trained fifteen women aged 24 to 35 years of age. All had breastfed at least one child. All were married with at least one child and the majority (75%) had received at least seven years of formal education. Almost all were subsistence farmers. The demographic characteristics of the peer counsellors are summarized in Table 2.

**Table 2:** Demographic characteristics of the trained peer counsellors

Age group in years	Number
20–24	3
25–30	7
31–35	4
Formal Education level	
Primary 1–4	0
Primary 5–7	3
Secondary level	12
Occupation	
Subsistence farmer	12
Other	3
Residence	
Village	14
Trading centre	1
Number of children	
1–3	6
More than 3	9
Marital status	
Married	15
Single	0

All selected mothers reported promptly and participated in all the sessions. Three mothers came with their breastfeeding babies one of whom also came with her younger sister to help her look after the baby during the training.

The peer counsellors initiated the election of group leaders responsible for time keeping, welfare, spiritual matters, information and a chairperson. They suggested norms for the workshop, such as strict time management, avoidance of unnecessary movement, respect for each other, cooperation, and participation in all the training activities.

The peer counsellors said they understood the content of the course; gained correct knowledge about breastfeeding and how to support mothers to exclusively breastfeed successfully. They said that most of their expectations were met by the end of training and were happy with the knowledge gained. A 28 year old peer counsellor said:

"Before this training, I thought that breast milk alone was not enough for my baby. Now I know that my milk is enough even for as long as 6 months. It will save me a lot of problems."

She went on:

"Mothers will be happy to realize that they have enough breast milk."

We observed that the peer counsellors understood the course content from the way they answered questions put to them and discussed issues during the training sessions.

During the trainers' meetings, it was noted that most peer counsellors were able to practice the counselling skills during the clinical practice sessions. All the peer counsellors completed the training and received certificates of attendance. They also requested a 'uniform' dress to wear for identification as peer counsellors for exclusive breastfeeding in the community.

During training it became apparent that the concept of counselling was a new way of sharing information which the peer counsellors found difficult to internalise. As we discussed the difference between information and advice, one of the peer counsellors observed,

"The consequences of giving advice are that the counsellor is blamed for the results; you may be reported to the political local chairperson if things go wrong. It may damage the work done by the counsellor because of lack of trust by the community. It is better to share with the mother and allow her to make a decision and the counsellor will not be blamed for any unfavourable consequences."

The peer counsellors felt a need to protect themselves from being blamed in case of problems.

### Cultural beliefs and practices related to breastfeeding

Some of the cultural beliefs discussed during training were possible obstacles to exclusive breastfeeding. A number of practices were believed to be dangerous for the child. For example:

"If you express milk to the ground, the baby will die."

Contrary to this belief, none of the participants reported having ever seen or heard of a child who died as a result of the mother expressing the milk. There were some divergent opinions concerning some practices, but consensus was reached on how to deal with each problem after discussion. We discussed ways of correcting the mistaken ideas in these beliefs without antagonizing the community. By the end of the training the peer counsellors were able to convey appropriate information using counselling skills they had acquired.

At the beginning of the training course for the health workers, they raised questions about breastfeeding similar to what the peer counsellors had wanted to know. They appreciated the knowledge obtained about breastfeeding from the training. All the seven health workers completed the course and received certificates of attendance.

### Evaluation of peer counselling

The peer counsellors were well received by the community. None of the peer counsellors was refused entry into any of the homes they visited to counsel mothers. The husbands also welcomed the idea of the peer counsellors helping their wives with breastfeeding. During focus group discussions with the husbands of the women who were offered counselling, all the participants hailed the introduction of peer counselling for breastfeeding to their community as a useful thing. The peer counsellors arranged their own visits at convenient times and each visited and helped, on average, 15 mothers who were either pregnant or breastfeeding. They continued to recruit pregnant mothers for follow-up and at each visit they would offer information about breastfeeding. For mothers planning to breastfeed, the peer counsellor offered to help the mother with breastfeeding after the birth.

One peer counsellor had recruited mothers at the local health centre during immunisation sessions. Others had talked to visiting mothers from other communities during functions such as funerals. Unfortunately such mothers could not be followed up. The peer counsellors mostly recruited the mothers by visiting them in their homes. Some mothers heard about the peer counsellors and called on them to discuss their breastfeeding problems. Of the 15 peer counsellors, nine reported in the monthly meetings that some mothers had come to them or sent messages to them asking for help with their breastfeeding problems.

The peer counsellors observed that many mothers had problems with positioning and attaching their babies at the breast. They were able to identify common breastfeeding problems as: cracked nipples, breast engorgement, mastitis and poor positioning at the breast. They further observed that most of these problems were eased by correct positioning of the baby at the breast. The peer counsellors had, also, observed that early introduction of cow's milk was widespread. They discovered that mothers resorted to this practice because they felt they did not have 'enough breast milk.' The issue of early introduction of cow's milk was mentioned by the peer counsellors at the monthly meetings. They reported that all the mothers they had visited were planning to, or had introduced cow's milk or thin cereal gruels to their babies before four months of age.

During the supervision visits, the supervisors noted that the peer counsellors seemed eager to share with the mothers everything they had learned in the workshop, regardless of the women's problem. For example, a mother with sore nipples and a two week-old baby got a lot of information on how to wean her baby.

As the peer counsellors visited mothers, neighbours would also come and join the discussion leading to a spill over of knowledge to more mothers. The counselled mothers were happy to have someone within their community helping them with breastfeeding problems and some reported that they were also helping their friends with similar problems. During the focus group discussions with the counselled mothers, they felt that the peer counsellors were like them, living under similar circumstances and easy to approach. A 22 year old mother said,

"Our peer counsellor is a jolly person, friendly and is very patient with us; she is easy to approach and has children of her own who are healthy. She has helped us and my sore nipples felt better after she helped me with positioning my baby at the breast."

During focus group discussions with the husbands of the counselled women, the breastfeeding mother, her mother in law and her husband were identified as key persons who can promote and support exclusive breastfeeding within the family.

One 32 year old man said,

"Husbands should love their wives; this helps the mother get enough breast milk for their child. In addition if you go home and find that your wife is not breastfeeding, it is your responsibility as a man to encourage her to breastfeed. I will give an example of animals. When an animal is well cared for and given adequate feeds it in turn produces a lot of milk, so when a mother is given adequate care and attention she is able to produce enough breast milk."

These focus group discussions further identified health care providers especially the midwives, both traditional birth attendants and professional midwives, as people who would support breastfeeding since they talk to mothers during pregnancy and eventually assist them during childbirth. They also help mothers with the initial positioning of the baby to the mother's breast. A 40 year old man said,

"....*it should be the midwives. This is because all pregnant mothers visit the midwife at one stage or another so they would be the right persons to counsel the mothers on breastfeeding. That is the work of the health workers they should be the ones to counsel the mothers. The good thing is that we have two types of midwives: we have the traditional birth attendants and the midwives in the health units. These two types should help the mothers to breastfeed. In addition these midwives should have breastfed children of their own because it is difficult to help somebody do something you have no experience in doing."*

### Follow-up and supervision of peer counsellors

The investigators of the study initially joined the district trainers to start them off on the supervision role. The peer counsellors appreciated the supervision visits and they shared their success and challenges. The supervisors observed the peer counsellors at work and reviewed their records. Two of the supervisors tended to take over from the peer counsellors during the supervisory visits, putting the peer counsellors in an observer role. This was pointed out during discussion at the end of the visit and the importance of supervisors taking on an observer role was emphasized. It was agreed the supervisors would correct any mistakes by the peer counsellors during the discussion at the end of the observation. One supervisor with a counselling background assumed the observer role more easily during the supervisory visits.

The peer counsellors were encouraged to use *Lusoga*, the local language, for reporting as some were trying to use English unsuccessfully.

The first group meeting was held four weeks after the training workshop, and twelve of the fifteen counsellors, attended. Three had personal and family issues to attend to. The peer counsellors presented their reports which were then discussed with the supervisors.

### Expectations of the peer counsellors

At the beginning of the training some peer counsellors were hoping to be trained as health workers while others wanted to learn how to improve breastfeeding of their babies. Some suggested that they receive uniforms to identify them in the community. The peer counsellors expressed a strong wish to be given bicycles to ease their mobility around the villages and a monthly allowance equivalent to US$10. Transportation was the most "felt need" identified by the peer counsellors. One peer counsellor said,

"When your child falls sick and you have that money, you have to use it to treat your child. What will you then use for transport to visit mothers who live some distance from your home?"

Another peer counsellor said,

"Transport was a problem; some of us had to walk long distances to visit mothers while carrying a baby on the back. At times it was very hot and on other days we were soaked by rain."

The peer counsellors were each given a bicycle for ease of movement during peer counselling visits. 

Lessons learnt from this study are summarised in Table 3.

## Discussion

This study showed that rural Ugandan women with modest formal education can be trained in breastfeeding counselling successfully. On returning to their communities, they were able to provide help and support to breastfeeding mothers to improve their breastfeeding technique and breastfeed exclusively. This is in agreement with what other studies have found [20-22].

The peer counsellors expressed a desire to learn more about breastfeeding at the beginning of the course. This was despite breastfeeding being culturally accepted and widely practiced in the community. The peer counsellors believed that breast milk alone was not enough for a baby up to the age of six months. A similar belief was also perceived at the lactation clinic of Mulago hospital in Uganda [31]. The training curriculum covered all the questions asked by the peer counsellors at the beginning of the course. This gave the peer counsellors the confidence that they would be able to answer questions posed by their peers. Since we did not administer pre- and post-test during training, our assessment of the knowledge they gained from the training is limited.

We also found that there are cultural and traditional beliefs and practices regarding breastfeeding which may influence the practice of exclusive breastfeeding negatively. Beliefs and practices related to expressing breast milk, use of colostrum together with understanding and managing breast conditions during breastfeeding may not be supportive of exclusive breastfeeding. Other studies have also highlighted traditional and cultural beliefs and practices related to breastfeeding that may negatively influence the practice of exclusive breastfeeding [7-9].

At the beginning of the training for health workers, they were asked what they expected to learn from the training course. A list of their expectations was made and it was interesting to note that most of the expectations of the health workers were similar to those of the peer counsellors at the beginning of training. This suggests that community women could perform as well as, or even better than the health workers in supporting mothers to exclusively breast feed their babies. However, we did not compare the performance of the two groups in this study.

The peer counsellors were also able to identify common breastfeeding problems in their communities. They appreciated the fact that the training they received had empowered them with skills to help the mothers overcome these problems. The commonly identified breastfeeding problems included "not enough breast milk", sore nipples and mastitis as well as identifying poor positioning of a baby at the breast. This was also reported in a previous hospital based study in Uganda [31].

We further observed that follow-up of the peer counsellors in their communities helped to motivate them so that they neither failed nor lost their confidence. Follow up supervision served as a way of addressing the challenges the peer counsellors met in their work and this was appreciated. It provided a mechanism for continued training for them as well sharing their experiences with each other and their supervisors. They were able to consult where they encountered difficulties. This interaction provided an avenue for the supervisors to re-enforce some information and skills which were observed to be deficient while observing the peer counsellors at work. Often the peer counsellors were able to suggest solutions during meetings which boosted their confidence further. This also added to their credibility with the mothers. This is similar to what Haider and colleagues reported in Bangladesh where peer counsellors felt that the supervisor's support and connection to the health facility gave them credibility with the mothers [20].

The acceptance of the peer counsellors by the community underscores the importance of involving the community right from the planning of the intervention. There was a general feeling, even among men, in this community that breastfeeding mothers needed more support. Many women meet challenges leading to interruption of exclusive breastfeeding with early introduction of other feeds or early cessation of breastfeeding altogether.

The peer counsellors were able to fit their counselling activities in their regular work routines which include work in the fields and household chores. One important issue raised by the peer counsellors concerned facilitation with transportation in the form of a bicycle. The bicycle is a common mode of transport in this area. This would be beneficial to the peer counsellors for their other needs, like taking their grain for milling or transporting sick children to the health unit, in addition to using them to visit and support breastfeeding mothers. Using a bicycle would mean less time spent visiting and supporting mothers. However, providing bicycles had financial implications for the study since they were not budgeted for during the planning of the study.

Being subsistence farmers most of these peer counsellors were having a new experience after being empowered with knowledge about breastfeeding. Most displayed a feeling of an "uplifted" status in the community which could explain why they wanted to get uniforms to identify them in their communities. A similar observation was made in one Glasgow study where the peer counsellors developed a "helpers' collective identity" [32]. However, we are not sure how wearing a uniform would impact on the program as the mothers in the community might regard them as some superior group rather than their peers.

We conducted this study in one sub-county in the district and its general application is difficult to predict. However, most rural populations seem to have similar characteristics in relation to socio-economic status and education levels [14].

It was observed that it was not easy to impart all the skills of counselling to these peer counsellors in the short time of training. They focused more on what is best for the counsellor rather than whether the counselling technique was the best for the breastfeeding mothers.

In conclusion, women from this rural community in Uganda were successfully trained as peer counsellors for exclusive breastfeeding. An important task for the trainers is to emphasize to the peer counsellors the importance of tailoring the information to each mother's need in addition to helping the peer counsellors with plenty of hands-on practice during the training.

They were accepted by their communities and were able to help and support their peers to successfully breastfeed their babies. The effect of peer counsellors on exclusive breastfeeding rates will be evaluated in a multi-centre community randomized controlled trial in Burkina Faso, Uganda, Zambia and South Africa which is in progress.

## Competing interests

The author(s) declare that they have no competing interests.

## Authors' contributions

JN designed and conducted the study as well as drafting the manuscript. JKT and TT designed the study and helped in drafting the manuscript. GN participated in design of the study and draft of manuscript. ÅS conducted the study and helped in draft of the manuscript. NS conducted the study and helped in draft of manuscript. All authors read and approved the final manuscript.

**Table 3:** Lessons learnt

**The Intervention**
• Training rural women as peer counsellors for support of exclusive breastfeeding is feasible
• Introducing an activity in a community can be a long process requiring multiple visits starting with the district down to the lowest level to ensure community involvement. This is important for the community to accept the peer counsellors.
• It is our impression that completely voluntary work is difficult to maintain in this rural Ugandan setting; discussions on how to compensate the peer counsellors for their time should be part of an exclusive breastfeeding intervention.
**The training**
• The trainers should be fluent in the local language in order to explain the concepts in a way that is easily understood by the peer counsellors.
• The training materials should be suitable for the local needs with appropriate illustrations and visual aids.
• Peer counsellors need more hands-on practice during training especially on the counselling skills using role-plays and more practice with real mothers during the training than provided in our course.
**Follow-up**
• The peer counsellors were able to use the knowledge acquired to help their peers in their communities and were easily accepted by their peers in the communities.
• For effective support supervision, supervisors need to be dedicated, for instance by being contracted on full time basis and paid.
• We also assessed the possibility of individual randomization in a larger study but concluded that it would be difficult to randomize individual mothers since they interact with each other and share their breastfeeding experiences; community randomization is probably a more feasible option.
